# Block copolymers from ionic liquids for the preparation of thin carbonaceous shells

**DOI:** 10.3762/bjoc.13.163

**Published:** 2017-08-16

**Authors:** Sadaf Hanif, Bernd Oschmann, Dmitri Spetter, Muhammad Nawaz Tahir, Wolfgang Tremel, Rudolf Zentel

**Affiliations:** 1 Institute for Organic Chemistry, University of Mainz, Duesbergweg 10-14, 55128 Mainz, Germany

**Keywords:** block copolymer, carbon, ionic liquid, polymeric ionic liquid, RAFT polymerization

## Abstract

This paper describes the controlled radical polymerization of an ionic-liquid monomer by RAFT polymerization. This allows the control over the molecular weight of ionic liquid blocks in the range of 8000 and 22000 and of the block-copolymer synthesis. In this work we focus on block copolymers with an anchor block. They can be used to control the formation of TiO_2_ nanoparticles, which are functionalized thereafter with a block of ionic-liquid polymer. Pyrolysis of these polymer functionalized inorganic nanoparticles leads to TiO_2_ nanoparticles coated with a thin carbonaceous shell. Such materials may, e.g., be interesting as battery materials.

## Introduction

Ionic liquids (ILs) are organic salts. Most of them have a melting point below 100 °C [1–2]. These organic salts do not have the same structure like inorganic salts. This is due to the structure of the ion pairs. They are built of organic asymmetric cations, like imidazolium, pyridinium or alkylammonium and inorganic anions, such as halides, mineral acid anions, or polyatomic inorganic anions (PF_6_^−^, BF_4_^−^) [3]. Because of the steric hindrance, they are not able to build a strong lattice like inorganic salts. Therefore, not much energy is needed to overcome the lattice energy and melt the salt. Ion liquids are also called “green solvents”, because of their low vapor pressure, fire resistance and thermal stability [4]. Beside this, they have a high ionic conductivity, large heat capacity and good thermal and chemical stability [5]. Properties, like solubility can be varied easily by exchanging the anion. Ionic liquids are often used as an electrolyte or organic solvent. Furthermore, they are also used in catalysis or in organic synthesis. Due to their selective solubility for ions [6–8], they can be used to predetermine the presence of ions on surfaces, a property which is very important for electrochemical conversions or the uptake of ions into the crystal lattice [9].

Polymeric ionic liquids (PILs) are made of ionic liquids with a polymerizable group, like a vinyl or acrylate group. They build a new class of macromolecules with unique properties. Alternatively, it is possible to coordinate low molar mass ionic liquids to polymers by complexation of their anions to cyclodextrin side chains. This can have an influence on their lower critical solution temperature (LCST) [10–11]. Beside their use as organic solvent, they are applied as catalytic membranes, thermotropic liquid crystals [12], polymer electrolytes, ionic conductive materials, CO_2_ absorbing materials, microwave absorbing materials and porous materials [4]. Most of these polymers were synthesized by free radical polymerization. There are just few reports about controlled/living radical polymerization, like nitroxide-mediated polymerization (NMP), atom transfer radical polymerization (ATRP) and reversible addition–fragmentation chain transfer polymerization (RAFT) [2]. In general, by controlled radical polymerization techniques it is possible to prepare polymers with narrow polydispersity, controlled molecular weight and also well-defined block copolymers. Such block copolymers with ionic liquid blocks might enable to control the properties of PILs spatially. An interesting aspect of this might be (i) a reduction of the dimension of the ion conductivity in PIL block copolymers due to their demixed morphology or (ii) the control of ion conduction near surfaces, if PIL brushes are fixed to a surface [13]. This last example of a spatially restricted access of ions to a surface can be very interesting in combination with redox reactions [14–15], a case in which the accessibility of special ions to the surface is crucial. Another aspect where spatial control gets crucial is the locally directed formation of thin carbonaceous shells. As demonstrated by Yuan et al., PILs are suitable carbon precursors with high carbon yields and good electric conductivity [16]. There are many different morphologies of carbon achievable, like hallow carbon spheres [17], nanotubes, membranes and fibers [18]. Due to their charged nature the PILs show a low vapor pressure and are non-volatile, leading to high carbon yields [19]. Furthermore, PILs offer the possibility of selective doping of the carbon by the choice of the counter ion. Heteroatoms like nitrogen and phosphor can be incorporated into the carbonaceous shell to improve or enhance properties like catalytic and electronic conductivity [18,20–21].

Independently from the work on polymeric ionic liquids, thin shells of carbonaceous materials around inorganic nanoparticles have been intensively investigated recently [22–25]. This interest is related to the search for improved battery materials for the reversible storage of electricity. To further improve batteries in terms of energy and power density, current research activities are directed, for example, towards new electrode active materials like TiO_2_, ZnO, Si or LiFePO_4_ [26]. However, both electronic and ionic conductivity of these materials are typically rather low. To overcome this issue, the combination of nanostructuring and the incorporation of conductive carbon was shown to be a successful strategy [27]. While nanostructuring of inorganic particles increases the electrode/electrolyte contact area and allows an easier diffusion of the cations, the incorporation of electronic transport pathways allows an improved charging of the nanoparticles [27]. In this context carbonaceous secondary structures and coatings [27–28] can be applied to increase electronic conductivity. In addition, the surface reactivity of the nanosized particles in contact with the electrolyte is reduced. Recently, it could be shown that block copolymers with an anchor group could bind to inorganic nanoparticle surfaces, where a second polymer block could be converted into a conductive carbon shell, improving the properties of nanoparticles like TiO_2_ or ZnO with respect to the reversible storage of lithium or sodium ions [22–25]. Using a block copolymer with an anchor group to bind on the nanoparticle surface allows the formation of a homogenous and thin coating. So far, polyacrylonitrile has been used as a carbonizable block, but polymeric ionic liquids are attractive as well.

An approach to coat nanoparticles with either (i) a thin film of PILs or (ii) a homogeneous carbonaceous layer derived from ionic liquids requires – at first – a synthetic route to block copolymers, which possess besides an anchor block [29], a block of polymerized ionic liquid monomers. Such a route will be presented here.

## Results and Discussion

**Figure 1:**
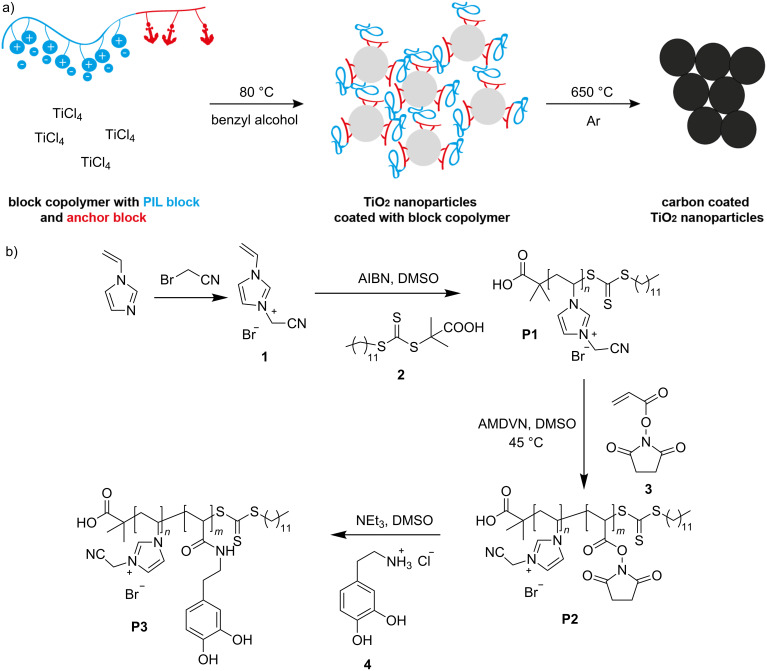
(a) Schematic illustration of the synthesis route of carbon coated TiO_2_ nanoparticles. (Left) in situ synthesis of the TiO_2_ nanoparticles with the block copolymer as a ligand on the surface followed by the pyrolysis of the particles resulting in homogenously coated nanoparticles. (b) Synthesis route for the preparation of the block copolymer, beginning from the monomer synthesis to the block copolymer and finally the post-polymerization modification step.

The schematic synthesis route to carbon-coated TiO_2_ nanoparticles using block copolymers is displayed in Figure 1a. The block copolymers containing an anchoring block and a carbonizable block should function – at first – as a ligand for the nanoparticle synthesis to produce polymer functionalized nanoparticles. The heat treatment at 650 °C of the hybrid material enables the conversion of the polymer shell into a carbon shell. The required block copolymers containing the carbonizable block and the anchoring block, which can bind onto the nanoparticle surface, was synthesized by RAFT polymerization as described in Figure 1b.

**Figure 2:**
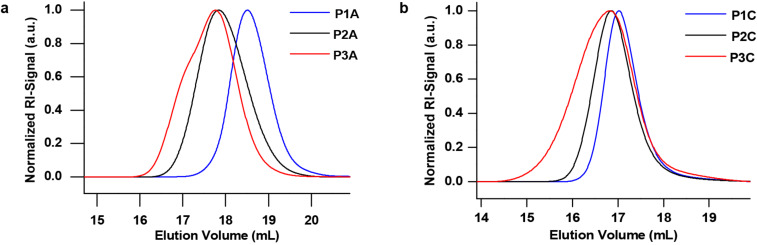
a) Size-exclusion chromatography of **P1A** (blue), **P2A** (black) and **P3A** (red) and b) size-exclusion chromatography of **P1C** (blue), **P2C** (black) and **P3C** (red) in hexafluoroisopropanol (HFIP). As expected, **P2** shows a shift towards higher molecular weight, which confirms the successful synthesis of the block copolymer. **P3** shows no further shift but a broader distribution, due to the dopamine group which interacts with the column material.

In a first step the PIL block is synthesized using 1-vinyl-3-cyanomethylimidazolium bromide (**1**) as an IL monomer, which was prepared following a literature procedure [16]. During this process the nitrogen atom in the imidazole ring in position 3 is quaternized. Monomer **1** was polymerized with 2-dodecylsulfanylthiocarbonylsulfanyl-2-methylpropionic acid (DMP, **2**) [30] as a chain transfer agent (CTA) and α,α’-azoisobutyronitrile (AIBN) as the initiator in the RAFT polymerization. Even though the synthesis of PILs by applying a controlled process has been reported to be difficult [1], we could obtain PILs in a controlled way by using a high ratio of initiator to CTA (1:2). Following this procedure we could vary the molecular weight of the PIL by variation of the CTA:monomer ratio and synthesize different block copolymers (see Table 1). The obtained polymers were characterized by size-exclusion chromatography (SEC), the elugrams are shown in Figure 2 and Figure S4 (Supporting Information File 1). The polymers described in our work have a narrow polydispersity index (PDI) varying from 1.11 (for the PIL block) up to 1.23 for the block copolymer. In order to show how controllable the polymerization of IL by RAFT polymerization is, we synthesized three block copolymers with different chain lengths for both the PIL block and the anchor block. For the PIL block we could synthesize short blocks, containing only 22 repeating units, as well as longer chain lengths consisting of 38 or 72 monomer units (as estimated by ^1^H NMR). The corresponding SEC elugrams (Figure 2 and Figure S4, Supporting Information File 1) reveal that the dispersity of the first block is quite narrow in all cases (PDI *<* 1.20). All the data regarding molecular weight and polydispersity are listed in Table 1. The average block length of the anchor group was kept constant with 20 repeating units (estimated by ^1^H NMR spectroscopy).

**Table 1:** Molecular weight and polydispersity of all synthesized polymers. **P1A–C** represents the PIL block. **P2A–C** represent the block copolymer and **P3A–C** the polymer after post-polymerization.

**P1**	*M*_n_ (g mol^−1^)	PDI		**P2**	*M*_n_ (g mol^−1^)	PDI		**P3**	*M*_n_ (g mol^−1^)	PDI
				
**P1A**	8 400	1.12		**P2A**	12 501	1.25		**P3A**	13 660	1.31
**P1B**	15 930	1.11		**P2B**	22 718	1.17		**P3B**	23 922	1.23
**P1C**	21 926	1.20		**P2C**	27 205	1.26		**P3C**	29 459	1.54

**Figure 3:**
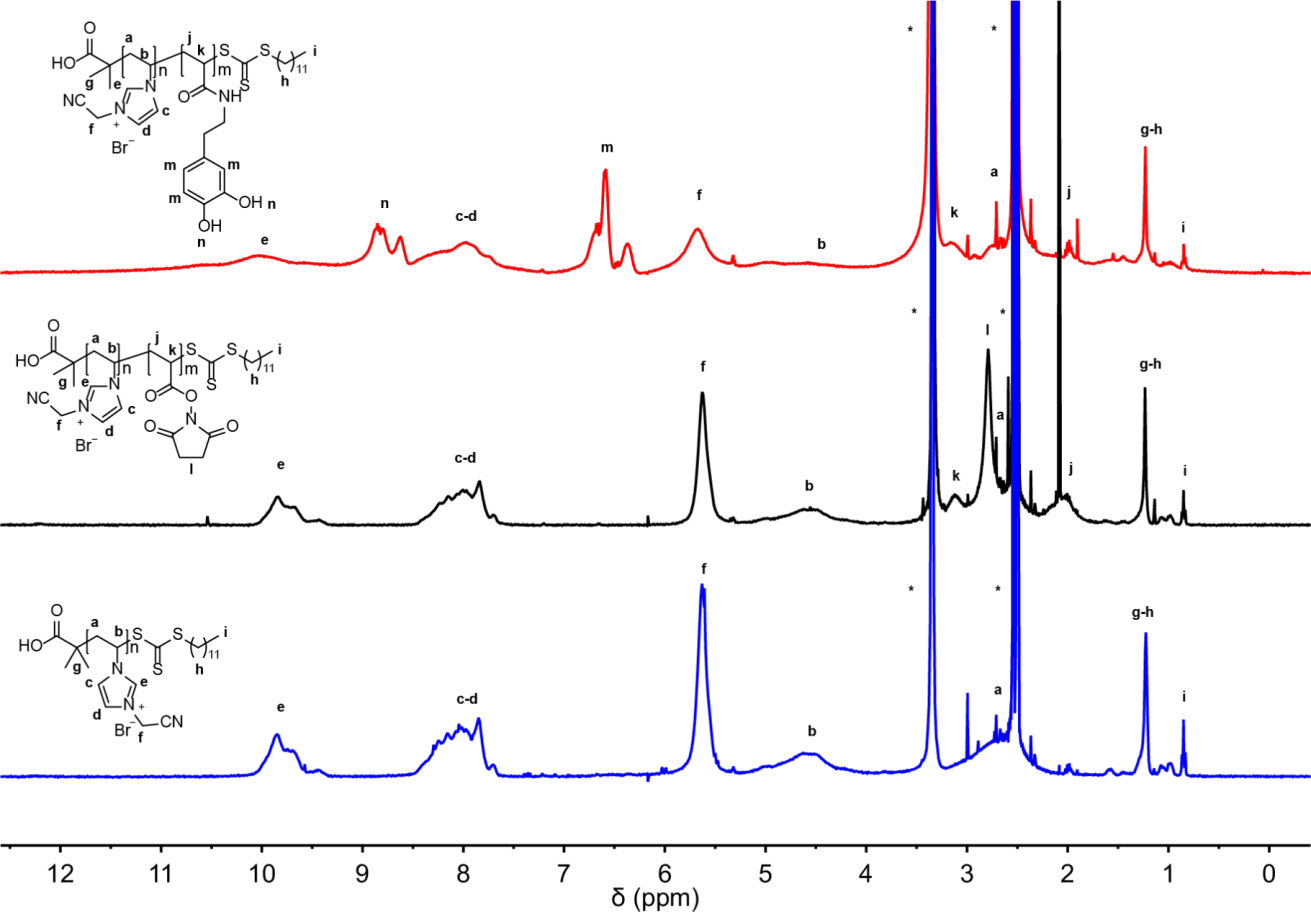
^1^H NMR spectrum of **P1**, **P2** and **P3**, all measured in DMSO-*d*_6_. In blue the spectrum of the PIL block is shown. The black spectrum belongs to the block copolymer with the reactive ester block. At 2.8 ppm a new shift can be seen, which is dedicated to the succinimide group in the reactive ester. The spectrum in red shows the polymer after the post-polymerization step. The shift at 2.8 ppm from the reactive ester disappeared. At 6.5 ppm and 8.8 ppm the chemical shifts from the dopamine group are shown.

All synthesized polymers were characterized by ^1^H NMR spectroscopy, which is shown in Figure 3. For the PIL block the spectrum is shown in blue. The resonance signals which occur at higher chemical shifts (7.8–9.8 ppm) belong to the protons in the imidazolium ring. The chemical shifts at 0.8 ppm and 1.2 ppm belong to the alkyl chain of the CTA, while the remaining signals are attributed to the polymer. The DOSY NMR spectrum (Figure S3 in Supporting Information File 1) proves that there is only one polymeric species. This excludes a mixture of homopolymers and demonstrates that block copolymers are obtained. The anchor block was thereby introduced in two synthetic steps. First, a block copolymerization using a reactive ester monomer was performed. Subsequently, the reactive ester block was aminolyzed to introduce dopamine (**4**) as the anchoring unit. Dopamine has been proven to coordinate well on transition metal oxide surfaces [29,31–32]. This route was chosen because dopamine cannot be polymerized in a radical process due to its phenolic structure that would act as an inhibitor. Hence we use the reactive ester chemistry by first introducing an active ester block, which can be easily substituted afterwards in a post-polymerization modification process. *N*-Acryloxysuccinimide (NAS, **3**) was chosen as a reactive ester because of its tolerance towards trace amounts of water present in DMSO, which is required for the block copolymerization as a polar solvent to solubilize the PIL macro-CTA. Optimized reaction conditions using 2,2-azobis(4-methoxy-2,4-dimethylvaleronitrile) (AMDVN) as an initiator, resulted in the successful block copolymerization. This was confirmed by ^1^H NMR spectroscopy after stirring for 20 hours at 45 °C. The broad signal which is typical for the NAS block can be observed at 2.8 ppm as shown in Figure 3. Another proof for the formation of a reactive ester block was given by IR spectroscopy. A new band can be observed at 1732 cm^−1^ and is assigned to the carbonyl group of the reactive ester (see Figure S5, Supporting Information File 1). In the last step the aminolysis of the reactive ester block with dopamine was performed, which leads also to a partial removal of the thioester end group. For this purpose a large excess of dopamine was applied. The ^1^H NMR spectrum in Figure 3 proves the successful conversion of the reactive ester to the corresponding amide. The NAS shift at 2.8 ppm vanished, while new shifts appeared at 6.5 ppm and in the range of 8.5–8.8 ppm corresponding to the aromatic ring of dopamine. This can be further confirmed by IR spectroscopy (Figure S6, Supporting Information File 1), where the NAS band disappeared, whereas a new band at 1647 cm^−1^ appears, which is assigned to the newly formed amide bond.

**Figure 4:**
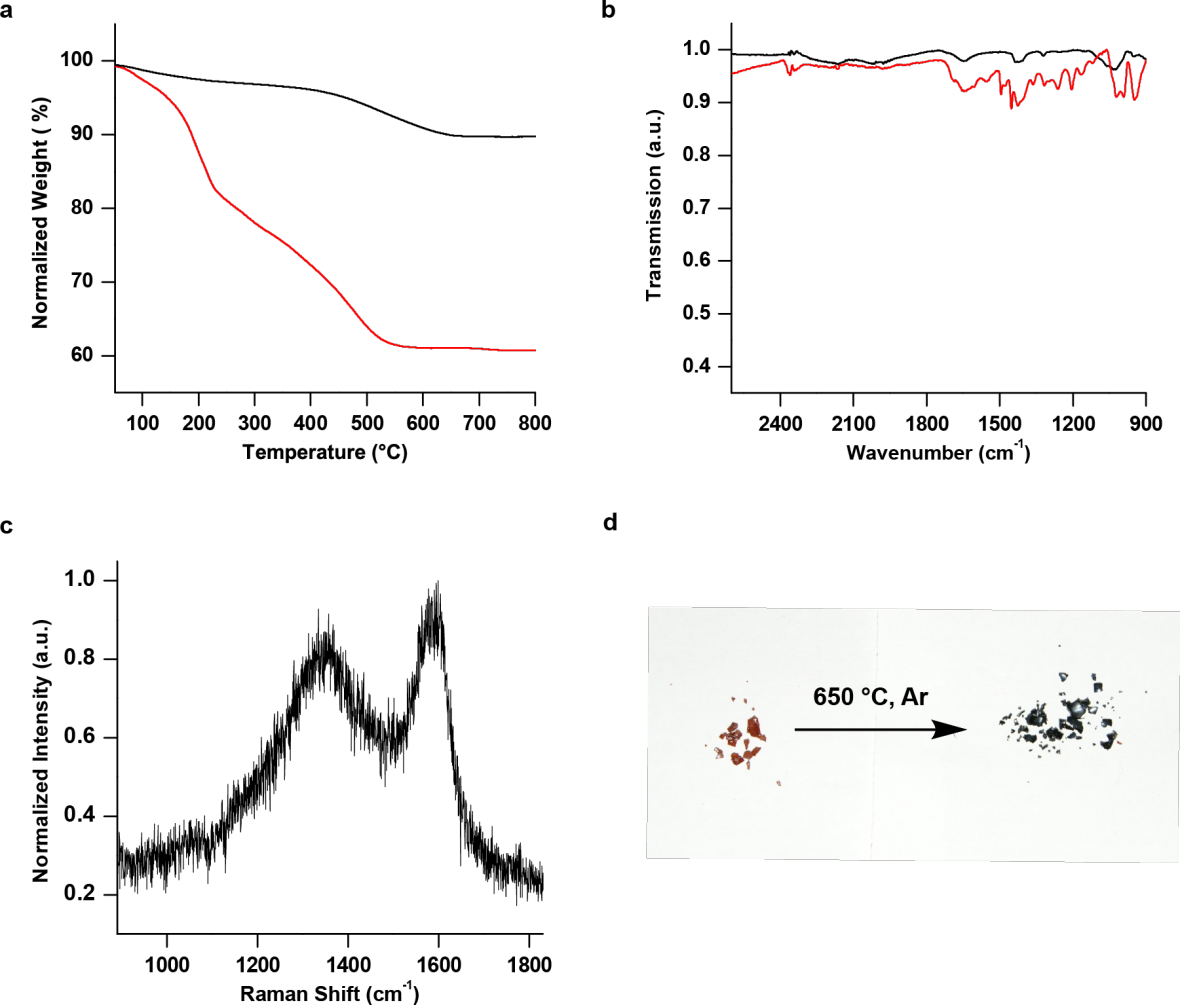
a) TGA measurement of the particles coated with block copolymer and particles coated with carbon, measured under oxygen atmosphere with a heating rate of 5 °C/min. In red the functionalized particles before pyrolysis. The weight loss up to 200 °C indicates the presence of the solvent (benzyl alcohol) which was used for the preparation of the particles. A mass loss of 20% can be observed. The black curve shows the functionalized particles after pyrolysis. b) IR spectrum of pure TiO_2_ particles (black) and the functionalized particles with the block copolymer on the surface (red). New bands are visible from 1685 cm^−1^ to 1166 cm^−1^ attributed to the block copolymer, showing their presence on the surface. c) Raman spectrum of pyrolyzed particles, showing the D-Band (1355 cm^−1^) and G-band (1584 cm^−1^), which proves the carbonaceous structure. d) Picture of the functionalized particles before (brown) and after pyrolysis (black).

**Figure 5:**
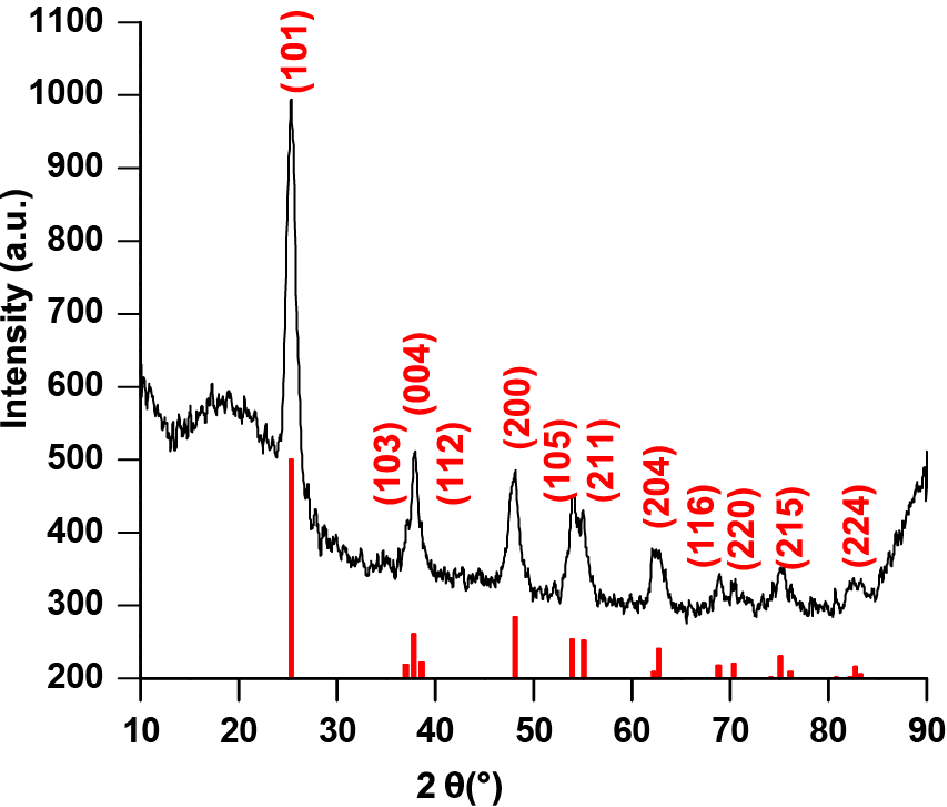
PXRD pattern of carbon-coated TiO_2_ particles.

The block copolymers **P1C**–**P3C** were used for the in situ synthesis of TiO_2_ particles [33–34]. Here, the block copolymer has several functions. It acts as a ligand during the nanoparticle synthesis avoiding the aggregation of nanoparticles, which would lower the surface area and increases the diffusion distances in the final particles for Na or Li ions. For the in situ nanoparticle synthesis TiCl_4_ was dissolved in benzyl alcohol and the block copolymer was added and stirred at 80 °C for 72 hours. The resulting brown suspension was precipitated using chloroform and hexane (1:3) and the precipitated product was centrifuged. The process was repeated three times to remove solvent and unbound ligand. The product was dried under vacuum at room temperature. To examine the content of ligands on the surface, thermogravimetric analysis was performed (TGA) after several centrifugation steps, as shown in Figure 4a. A total weight loss of 20% was determined. Although the particles were dried proper in high vacuum a shoulder around 200 °C shows up. This shoulder belongs to benzyl alcohol, which was used as a solvent for the synthesis. As a rough estimate for the weight loss of the coordinated polymer only the weight loss above 240 °C is considered to 20%. For the carbonization process the hybrid material was pyrolyzed in argon atmosphere and heated up to 650 °C. The application of higher temperatures (above 700 °C) is not advisable. Due to the use of TiO_2,_ phase transitions of the anatase TiO_2_ might occur, which leads to a mixture of anatase and rutile TiO_2_. XRD measurements (Figure 5) show that under the applied conditions, the pyrolyzed nanoparticles still contain TiO_2_.

In addition, a macroscopic color change of the hybrid material can be observed. As-synthesized TiO_2_ nanoparticles coated with the block copolymer looks brown due to the bound catechol. However, the color turns black after the pyrolysis (Figure 4d) indicating the presence of carbon material. This was proven by Raman spectroscopy revealing typical carbonaceous bands, such as the G-band at 1584 cm^−1^ and the D-band at 1355 cm^−1^, which is shown in Figure 4c. Furthermore, the residual carbonaceous content was determined by TGA, where the weight loss decreases from 20% (for the block copolymer coated particles) to 10% for the carbon coated particles (Figure 4a).

**Figure 6:**
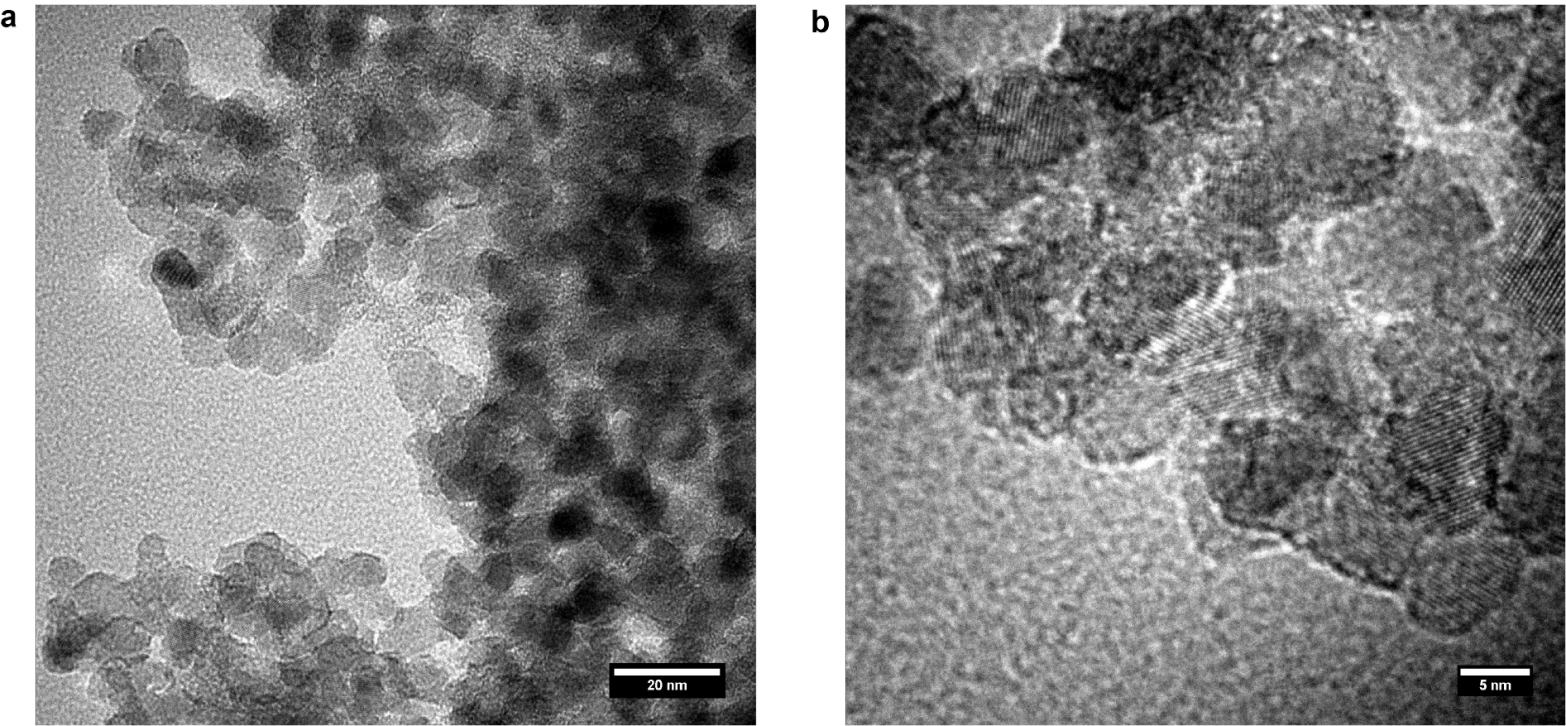
TEM images of the carbon coated TiO_2_ nanoparticles.

The resulting particles were also characterized by transmission electron microscopy (TEM), and corresponding images are shown in Figure 6a and 6b. The average particle diameter is ≈8 nm. Figure 6b shows nanoparticles sheathed and connected through lattices which might also help to provide longer paths for electrons to travel within the electrode. Summarizing, the Raman spectrum, the TGA measurements and the TEM images proves the success of the formation of a thin coating around the TiO_2_ particles. Currently, we are investigating the application of the hybrid material in batteries.

## Conclusion

In conclusion, we were able to synthesize well-defined block copolymers containing a PIL block and a reactive ester block. Besides, we showed the post-polymerization modification of these polymers, while remaining the block copolymer structure and simultaneously introducing an anchor group. Afterwards, we showed the successful in situ synthesis of TiO_2_ particles with the block copolymer as a ligand on the surface. Raman spectroscopy and TEM images show that PILs are suitable carbon precursors and the herein introduced materials can be further applied as anode material in lithium or sodium ion batteries.

## Experimental

All chemicals were acquired from commercial sources (Acros or Sigma-Aldrich) and used without further purification. Synthesis and structural characterization: NMR spectroscopy was applied with a Bruker ARX 400 spectrometer. Fourier-transform infrared (FTIR) spectroscopy was conducted on a Jasco FT/IR 4100 spectrometer with an attenuated total reflectance (ATR) unit. The SEC measurements were carried out at 40 °C with a solution of HFIP with 3 g L^−1^ K^+^TFA^−^ as eluent. Modified silica was used as stationary phase and a refractive index detector, JASCO G1362A RID, was used. Poly(methyl methacrylate) (PMMA) was used as calibration standard. TGA was performed with a Perkin Elmer Pyris 6 instrument with an oxygen flow. Raman spectroscopy was conducted with Horiba Jobin Y LabRAM HR spectrometer with a frequency doubled neodymium-doped yttrium aluminum garnet (Nd:YAG) laser. X-ray diffraction was performed on a Siemens D 5000 diffractometer equipped with Cu Kα radiation source (wavelength of 1.54056 Å) for both as synthesized as well as carbon coated TiO_2_ nanoparticles. TEM samples were prepared by dispersing the sample in ethanol and drop casting on 300 mesh carbon coated copper grids. The images were captured with a transmission electron microscope, a Tecnai G2 Spirit with an acceleration voltage of 120 kV.

**Synthesis of PIL:** The IL and also DMP which was used as chain transfer agent, were synthesized as already described in the literature [16,30]. For the RAFT polymerization the IL monomer (1 equiv), DMP (0.05 equiv for **P1A**, 0.02 equiv for **P1B**, 0.013 equiv for **P1C**) and the initiator AIBN (0.025 equiv for **P1A**, 0.01 equiv for **P1B**, 6.5·10^−3^ equiv for **P1C**) were mixed together and dissolved in DMSO, followed by three freeze-pump-thaw cycles. The reaction mixture was stirred for 20 h at 70 °C. Afterwards the mixture was purified by precipitation in acetone. ^1^H NMR (400 MHz, DMSO-*d*_6_) δ (ppm) 9.85 (m, C-2 of imidazolium ring), 8.01 (m, C-4 and C-5 of imidazolium ring), 5.63 (s, CH_2_CN), 4.62 (br, polymer backbone), 2.91 (m, polymer backbone), 1.23 (m, CTA dodecyl chain), 0.85 (t, dodecyl-CH_3_ of CTA); FTIR ν: 2973 (w), 2255 (w), 1626 (m), 1553 (s), 1425 (m), 1159 (s), 1019 (m), 748 cm^−1^ (w).

**Synthesis of P (IL-*b*-NAS):** PIL was used as macro-CTA. Together with NAS (20 equiv) and 2,2-azobis(4-methoxy-2,4-dimethylvaleronitrile) (0.2 equiv) PIL was dissolved in DMSO. After three freeze-pump-thaw cycles the mixture was stirred at 40 °C for 20 h. Afterwards the polymer was worked up by precipitation in acetone. ^1^H NMR (400 MHz, DMSO-*d*_6_) δ (ppm) 9.85 (m, C-2 of imidazolium ring), 7.85 (m, C-4 and C-5 of imidazolium ring), 5.64 (s, CH_2_CN), 4.51 (br, polymer backbone), 2.91 (m, polymer backbone), 2.80 (s, CH_2_-CH_2_ of NAS), 1.23 (m, CTA dodecyl chain), 0.85 (t, dodecyl-CH_3_ of CTA); FTIR ν: 2969 (w), 2255 (w), 1808 (m), 1732 (s, C=O, reactive ester), 1553 (s), 1204 (m), 1161 cm^−1^ (m); SEC (eluent: HFIP): 23 098 g mol^−1^, PDI = 1.17.

**Synthesis of P (IL-*b*-DAAM):** P (IL-*b*-NAS) (1 equiv) and lithium bromide (50 equiv) were dissolved in DMSO in a Schlenk flask. Dopamine hydrochloride (50 equiv) and triethylamine (50 equiv) were also dissolved in DMSO. The two solutions were combined and stirred overnight at 50 °C. For work-up, the polymer was precipitated in acetone. ^1^H NMR (400 MHz, DMSO-*d*_6_) δ (ppm) 9.97 (m, C-2 of imidazolium ring), 8.85 (br, OH of dopamine), 7.94 (m, C-4 and C-5 of imidazolium ring), 6.58–6.36 (br, ArH of dopamine), 5.66 (s, CH_2_CN), 4.58 (br, polymer backbone), 3.15 (br, polymer backbone), 2.91 (m, polymer backbone), 1.23 (m, CTA dodecyl chain), 0.85 (t, dodecyl-CH_3_ of CTA); FTIR ν: 2969 (w), 2255 (w), 1691 (m), 1645 (m, C=O, amide of dopamine), 1553 (s), 1434 (m), 1160 (m), 1020 cm^−1^ (m); SEC (eluent: HFIP): 23 180 g mol^−1^, PDI = 1.22.

**Synthesis of in situ functionalized TiO_2_ nanoparticles:** 400 mg of catechol containing polymeric ligand was dissolved in 10 mL of DMSO (Sigma-Aldrich) and added to 70 mL of benzyl alcohol (Acros). The content of the flask was heated to 80 °C. The solution was degassed and filled with argon using a Schlenk line. The process was repeated three times. To this argon filled solution 3.2 mL of TiCl_4_ was slowly injected under vigorous stirring. The dark red solution was kept at 80 °C for 72 hours while constantly stirring at 750 rpm. The resulting brown suspension was precipitated using CHCl_3_ and hexane (1:3) and the precipitated product was centrifuged. The process was repeated three times to remove the solvent and unbound ligand. The product was dried under vacuum at room temperature.

**Pyrolization of as-functionalized TiO_2_ nanoparticles:** All samples were pyrolyzed using the same conditions. 40 mg of the as-functionalized TiO_2_ nanoparticles were filled in a corundum boat, which was placed in a tube furnace. The heating rate was 5 °C/min up to a temperature of 650 °C, which was held for 1 h under a constant flow of argon. After that, the samples were cooled down naturally.

## Supporting Information

File 1 Additional spectra.
