# Supplementary material for: Block copolymers from ionic liquids for the preparation of thin carbonaceous shells
Source: Beilstein J Org Chem. 2017 Aug 16;13:1693–701. doi: 10.3762/bjoc.13.163 (PMC5564275; doi:10.3762/bjoc.13.163)
Supplement: File 1 — Additional spectra. [file Beilstein_J_Org_Chem-13-1693-s001.pdf]

Supporting Information

for

Block copolymers from ionic liquids for the preparation of thin carbonaceous shells

Sadaf Hanif, Bernd Oschmann, Dmitri Spetter, Muhammad Nawaz Tahir, Wolfgang Tremel and Rudolf Zentel*

Address: Institute for Organic Chemistry, University of Mainz, Duesbergweg 10-14, 55128 Mainz, Germany

Email: Prof. R. Zentel* - zentel@uni-mainz.de

*Corresponding author

Additional spectra

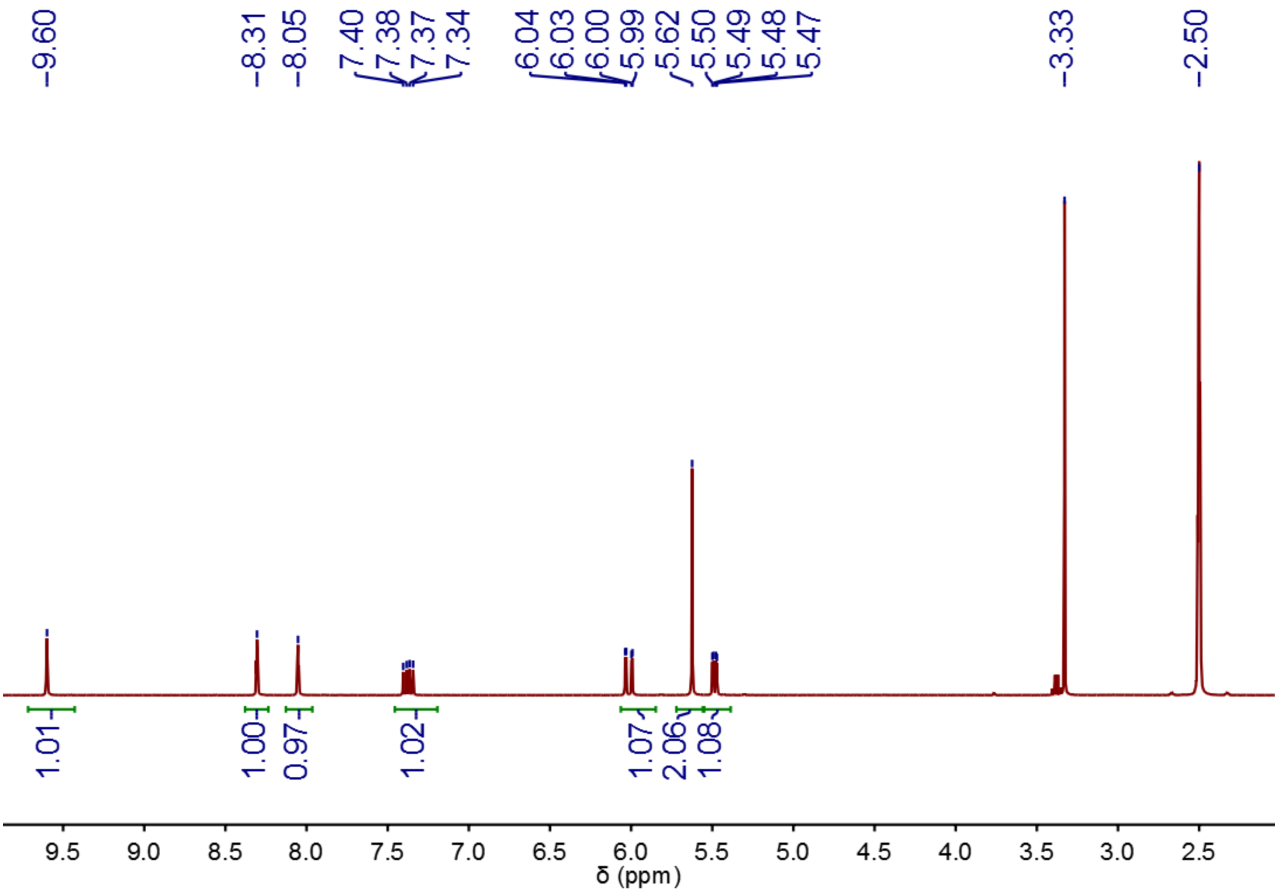

Figure S1: ^{1}H NMR of 1-vinyl-3-cyanomethylimidazolium bromide, measured in DMSO-d_6.

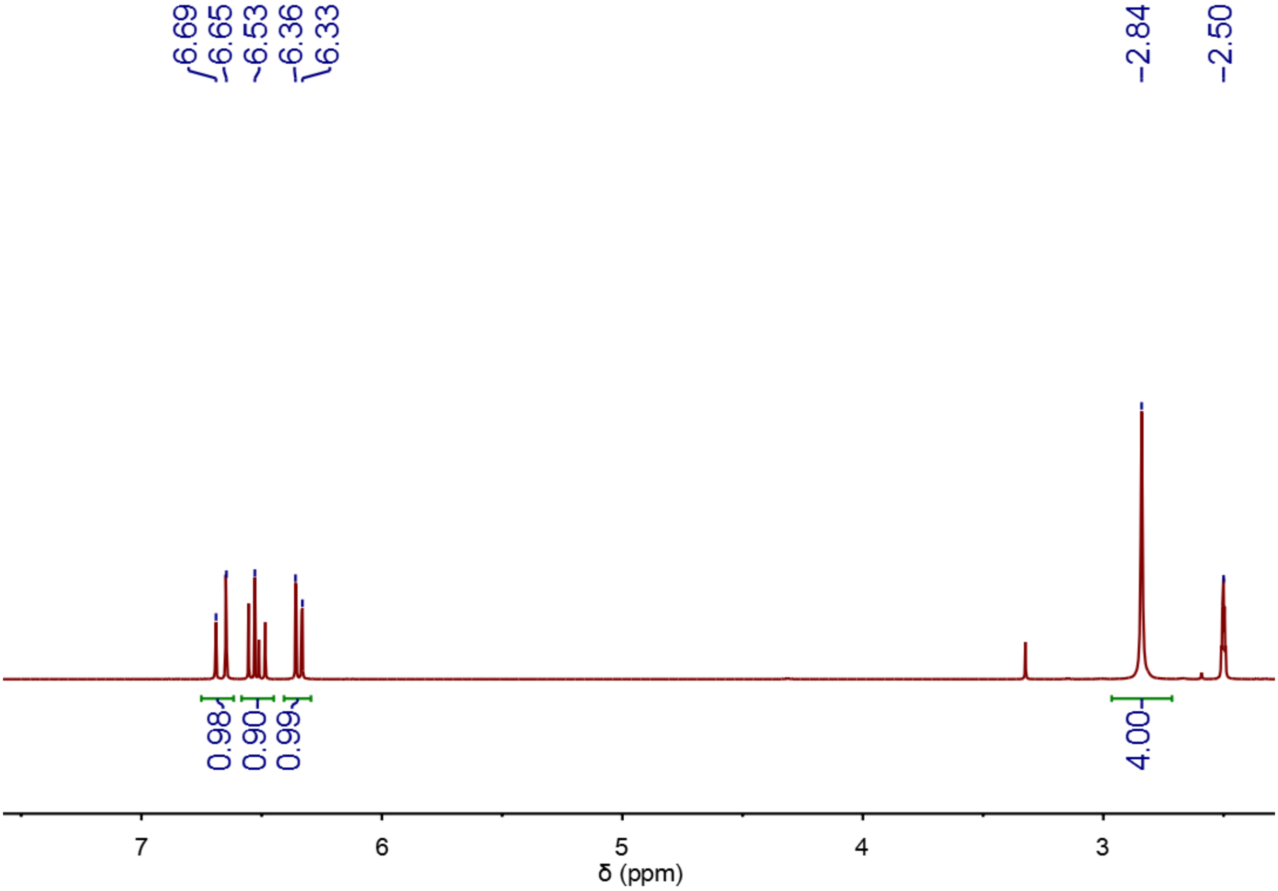

Figure S2: H NMR of NAS, measured in DMSO-d_6.

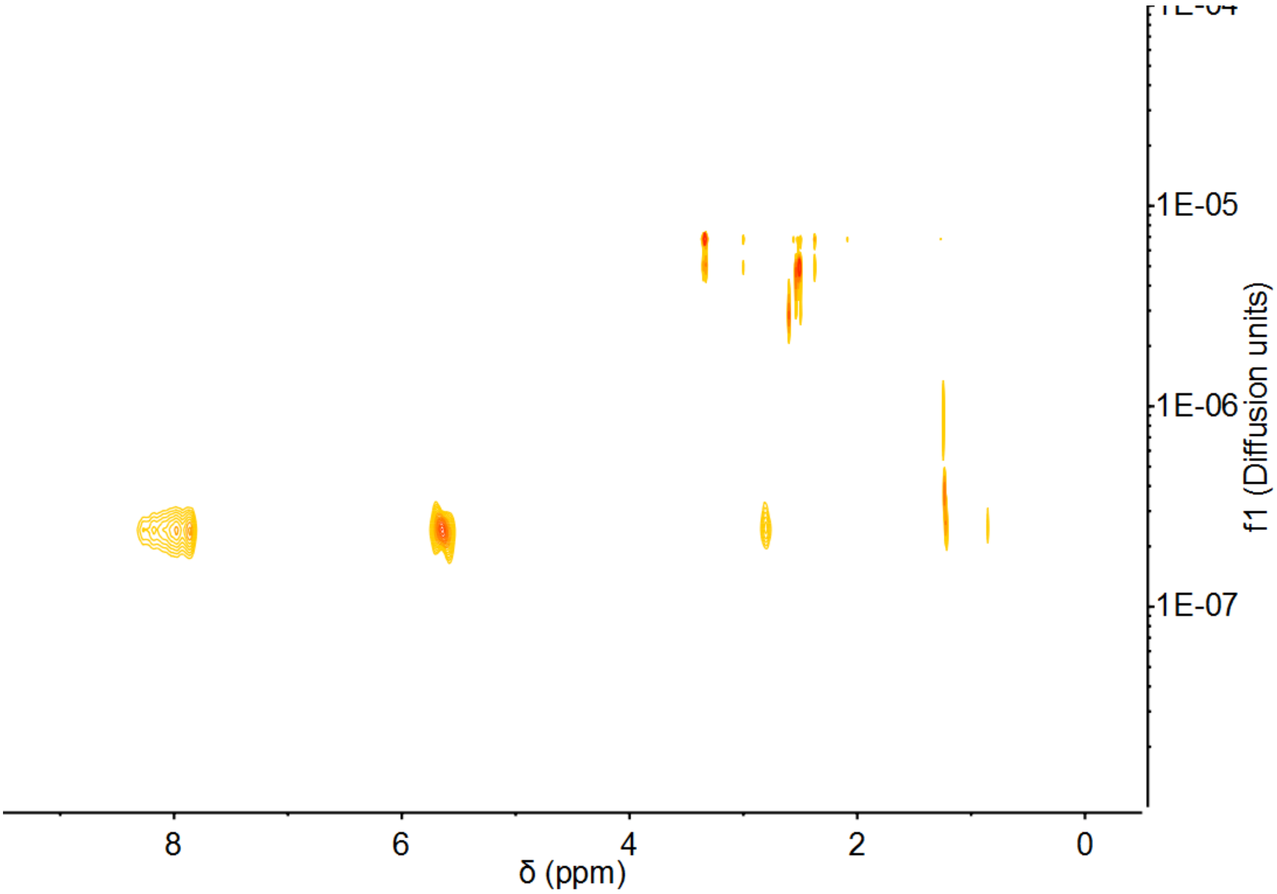

Figure S3: DOSY of the block copolymer **PB**. The spectrum shows just one polymeric species. DMSO and traces of water are also apparent, since the spectrum was measured in DMSO-d_6.

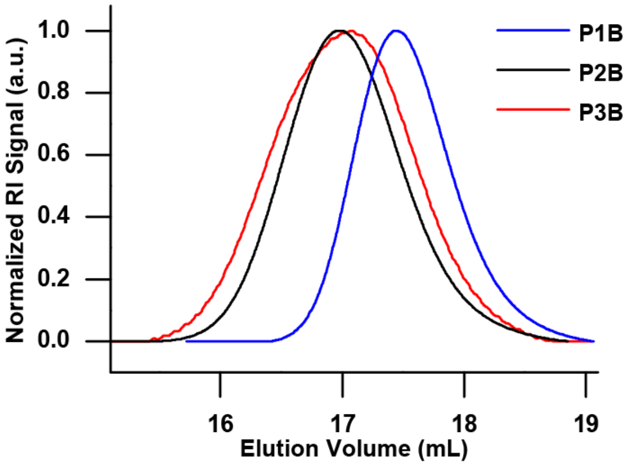

Figure S4: SEC of **P1B** (blue), **P2B** (black), **P3B** (red) measured in HFIP.

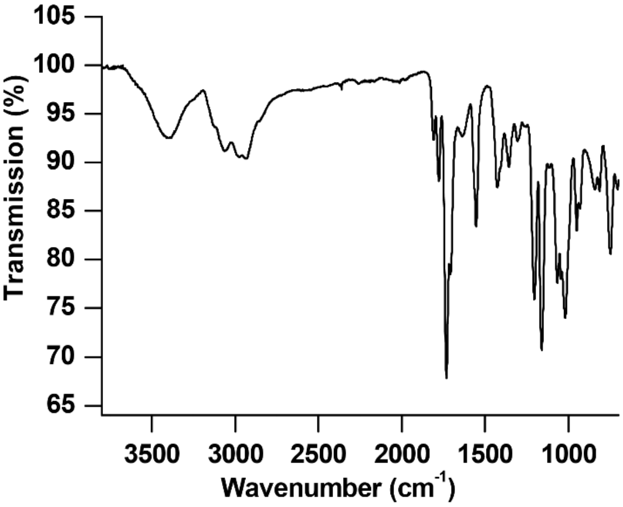

Figure S5: IR spectrum of the block copolymer **PB**.

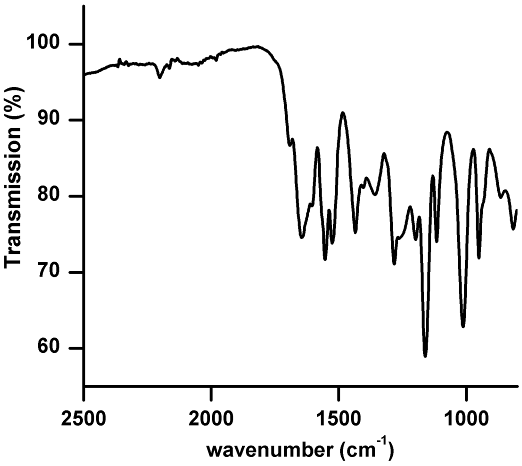

Figure S6: IR spectrum of the block copolymer after post polymerization modification (**PC**).
